# Effect of drugs on bone mineral density in postmenopausal osteoporosis: a Bayesian network meta-analysis

**DOI:** 10.1186/s13018-021-02678-x

**Published:** 2021-08-27

**Authors:** Filippo Migliorini, Nicola Maffulli, Giorgia Colarossi, Jörg Eschweiler, Markus Tingart, Marcel Betsch

**Affiliations:** 1 Department of Orthopaedic, Trauma, and Reconstructive Surgery, RWTH Aachen University Hospital, Pauwelsstraße 30, 52074 Aachen, Germany; 2 Department of Medicine, Surgery and Dentistry, University of Salerno, Via S. Allende, 84081 Baronissi, Salerno, Italy; 3 Queen Mary University of London, Barts and the London School of Medicine and Dentistry, Centre for Sports and Exercise Medicine, Mile End Hospital, 275 Bancroft Road, London, E1 4DG England; 4 School of Pharmacy and Bioengineering, Keele University Faculty of Medicine, Thornburrow Drive, Stoke on Trent, England; 5 Department of Orthopaedics and Trauma Surgery, University Hospital Mannheim, Medical Faculty of the University Heidelberg, Mannheim, Germany

**Keywords:** Osteoporosis, Bone mineral density, Drugs, Denosumab

## Abstract

**Background:**

Osteoporosis affects mostly postmenopausal women, leading to deterioration of the microarchitectural bone structure and low bone mass, with an increased fracture risk with associated disability, morbidity and mortality. This Bayesian network meta-analysis compared the effects of current anti-osteoporosis drugs on bone mineral density.

**Methods:**

The present systematic review and network meta-analysis follows the PRISMA extension statement to report systematic reviews incorporating network meta-analyses of health care interventions. The literature search was performed in June 2021. All randomised clinical trials that have investigated the effects of two or more drug treatments on BMD for postmenopausal osteoporosis were accessed. The network comparisons were performed through the STATA Software/MP routine for Bayesian hierarchical random-effects model analysis. The inverse variance method with standardised mean difference (SMD) was used for analysis.

**Results:**

Data from 64 RCTs involving 82,732 patients were retrieved. The mean follow-up was 29.7 ± 19.6 months. Denosumab resulted in a higher spine BMD (SMD −0.220; SE 3.379), followed by pamidronate (SMD −5.662; SE 2.635) and zoledronate (SMD −10.701; SE 2.871). Denosumab resulted in a higher hip BMD (SMD −0.256; SE 3.184), followed by alendronate (SMD −17.032; SE 3.191) and ibandronate (SMD −17.250; SE 2.264). Denosumab resulted in a higher femur BMD (SMD 0.097; SE 2.091), followed by alendronate (SMD −16.030; SE 1.702) and ibandronate (SMD −17.000; SE 1.679).

**Conclusion:**

Denosumab results in higher spine BMD in selected women with postmenopausal osteoporosis. Denosumab had the highest influence on hip and femur BMD.

**Level of evidence:**

Level I, Bayesian network meta-analysis of RCTs

## Introduction

Osteoporosis is common in postmenopausal women, with microarchitectural deterioration and low bone mass. Approximately, 19% of men and 30% of women in Europe and in the USA are at risk for osteoporosis, and annually around 9 million osteoporosis associated fractures occur [1]. Osteoporosis-associated fractures result in increased disability, mortality and health-care costs, and therefore the treatment and prevention of osteoporotic fractures carries significant clinical and public health importance [2].

Current approved pharmacological treatments for postmenopausal osteoporosis can be divided into anti-resorptive and anabolic medications [3]. Briefly, anti-resorptive drugs reduce bone resorption, whilst anabolic drugs increase bone formation. The most commonly prescribed agents are anti-resorptive drugs, which include bisphosphonates (BP) (e.g. alendronate, risedronate, zoledronic acid, ibandronate, etidronate), selective oestrogen receptor modulators (SERM) (e.g. raloxifene) and the RANK-ligand inhibitor (e.g. denosumab).

BP were discovered during the search for pyrophosphonate analogues, attempting to benefit from the inhibitory effects of pyrophosphates on calcification [4]. BP work by inhibiting the enzyme farnesyl pyrophosphonate synthase in osteoclasts, influencing their affinity for bone mineral uptake [5, 6]. During early treatment, SERMs decrease bone remodelling by about 20-30%, and thereby result in a modest transitory increase in bone mineral density (BMD) [7]. However, during prolonged therapy, SERMs lead to a decline in BMD, which may account for the only modest reduction in vertebral fracture risk [7].

Denosumab is a monoclonal antibody against the receptor activator of nuclear factor-kappa B ligand (RANK-ligand), a regulator of osteoclast development. By blocking the RANK-ligand with denosumab the activity, survival and recruitment of osteoblast are inhibited.

Anabolic osteoporosis drugs, such as teriparatide, are usually reserved for patients with severe and established osteoporosis. Both medications lead to an increase in trabecular thickness and improved trabecular microstructure via the teriparatide (PTHR1) receptor [8, 9]. Finally, romosozunab is a novel sclerostin antibody recently approved for the treatment of osteoporosis. Romosozunab has antifracture and anabolic efficacy, increasing bone formation and decreasing bone resorption [10, 11].

Network analysis may provide clinically relevant evidence in the absence of randomised controlled trials (RCTs) comparing relevant pharmaceutical treatments for osteoporosis. Therefore, we conducted this network meta-analysis comparing the effects of nine osteoporosis drugs and their effects on BMD in patients with postmenopausal osteoporosis.

## Methods

### Search strategy

The present systematic review and network meta-analysis follows the PRISMA extension statement for reporting of systematic reviews incorporating network meta-analyses of health care interventions [12]. The follow algorithm guided the preliminary search:

- P (population): postmenopausal osteoporosis
- I (intervention): medical treatments
- C (comparison): denosumab, raloxifene, teriparatide, alendronate, risedronate, zoledronate, ibandronate, etidronate, strontiumranelate
- O (outcomes): BMD

### Data source and extraction

The literature search was performed by two independent authors (FM; GC). In June 2021, the databases search started. The search on PubMed was performed with the following string: osteoporosis [All Fields] AND (bone [All Fields] OR endocrinology [All Fields]) AND (postmenopausal [All Fields] OR treatment [All Fields] OR management [All Fields] OR spine [All Fields] OR femur [All Fields] AND hip [All Fields] OR BMD [All Fields]) AND (mineral density [All Fields] OR Bisphosphonates [All Fields] OR Denosumab [All Fields] OR Raloxifene [All Fields] OR Teriparatide [All Fields] OR Alendronate [All Fields] OR Risedronate [All Fields] OR Zoledronate [All Fields] OR Ibandronate [All Fields] OR Etidronate [All Fields] OR Calcium [All Fields] OR Vitamin D [All Fields] OR PTH [All Fields] OR osteoblast [All Fields] OR osteoclast [All Fields]) AND management [All Fields] OR therapy [All Fields]. The same search strings were used to search Google Scholar, Embase and Scopus. The resulting titles and subsequent abstracts were screened by the same two authors. If they matched the topic, the article full-text was accessed. A cross reference of the bibliographies was also performed. Disagreement was debated and solved by a third senior author (NM).

### Eligibility criteria

All the randomised clinical trials (RCTs) investigating the effects of two or more drug treatments on BMD for postmenopausal osteoporosis were accessed. Given the authors language capabilities, articles in English, German, Italian, French and Spanish were eligible. Only levels I and II RCTs according to the Oxford Centre of Evidence-Based Medicine [13] were considered. Only articles reporting quantitative data under the outcomes of interest and articles with a minimum 12 months follow-up were considered. Studies treating patients with calcium and vitamin D without any other drugs were not included. Studies reporting data on patients with iatrogenic-induced menopause were not included, as well as those treating paediatric and/or adolescent patients. Studies on patients undergoing immunosuppressive therapies or organ transplantation were also not considered. Studies reporting data on patients with malignancies or pathological bone diseases other than osteoporosis were not included. Studies reporting data on mixed treatments or taking advantage from adjuvants were excluded. Editorials, registries, comments, expert opinions and reviews were not eligible. Animals or in vitro studies were also not eligible. Missing data under the outcomes of interest warranted the exclusion from this study.

### Outcomes of interest

Two authors (FM; GC) performed data extraction. Study generalities (author, year, journal, duration of the follow-up) and patient baseline demographic information were collected: number of samples and related mean age, percentage of female, mean bone mass index (BMI) and mean BMD (overall, spine, hip, femur neck). The following drugs were considered in the analyses: denosumab, raloxifene, teriparatide, alendronate, risedronate, zoledronate, ibandronate and etidronate. The outcome of interest was BMD at last follow-up.

### Methodology quality assessment

The methodological quality assessment was performed by two authors (FM; GC). The risk of bias summary tool of the Review Manager Software (The Nordic Cochrane Collaboration, Copenhagen) was used for evaluation. The following risk of bias was assessed: selection, detection, attrition and other source of bias.

### Statistical analysis

The statistical analyses were performed by the main author (FM). Baseline comparability was assessed through the IBM SPSS software. The analysis of variance (ANOVA) was used for analysis, with *P* values > 0.1 was considered satisfactory. The STATA Software/MP, Version 14.1 (StataCorporation, College Station, Texas, USA) was used for the statistical analyses. The NMA was performed through the STATA routine for Bayesian hierarchical random-effects model analysis. The placebo treatment was used as reference group. The inverse variance method was used for analysis, with standardised mean difference (STD) and standard error (SE) effect measures. The overall inconsistency was evaluated through the equation for global linearity via the Wald test, with *P* values< 0.05 indicating statistically significant inconsistency. Otherwise, if *P* > 0.05 the null hypothesis cannot be rejected, and the consistency assumption could be accepted at the overall level of each treatment. Both confidence (CI) and percentile (PrI) intervals were set at 95%. Edge plot, interval plots and funnel plots were obtained and evaluated.

## Results

### Search result

**Fig. 1:**
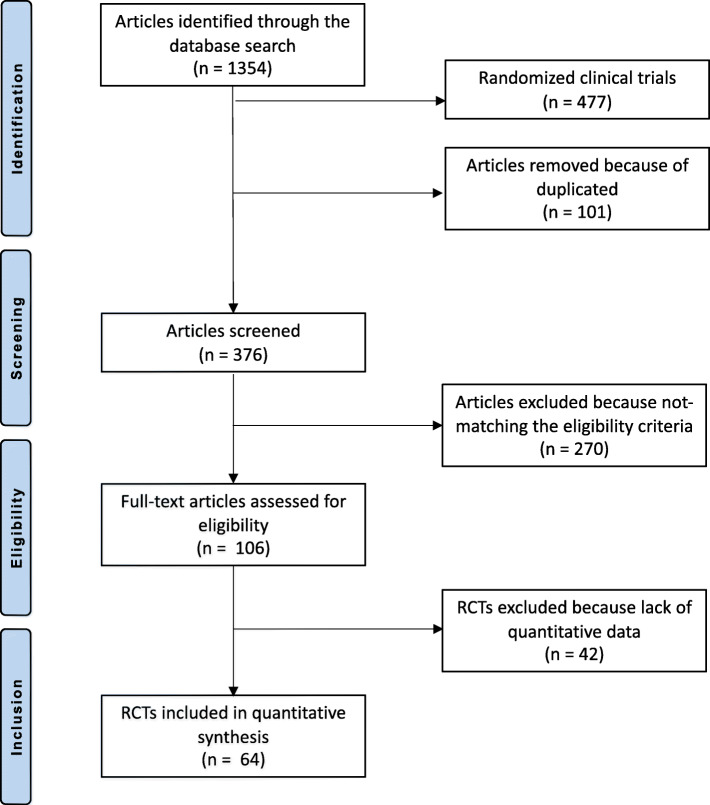
Flow chart of the literature search

The primary literature search resulted in 1354 articles. Of them, 477 were RCTs. A further 101 were removed because duplicated. Additional 270 articles were excluded because of the study design (*N* = 26), non-clinical studies (*N* = 34), glucocorticoid-induced osteoporosis (*N* = 51), treatment of bone malignancies (*N* = 56), language limitations (*N* = 12) and others (*N* = 91). A further 42 articles were excluded because it did not report quantitative data under the outcomes of interests. Finally, 64 RCTs were included for analysis. The literature search results are shown in Fig. 1.

### Methodological quality assessment

**Fig. 2:**
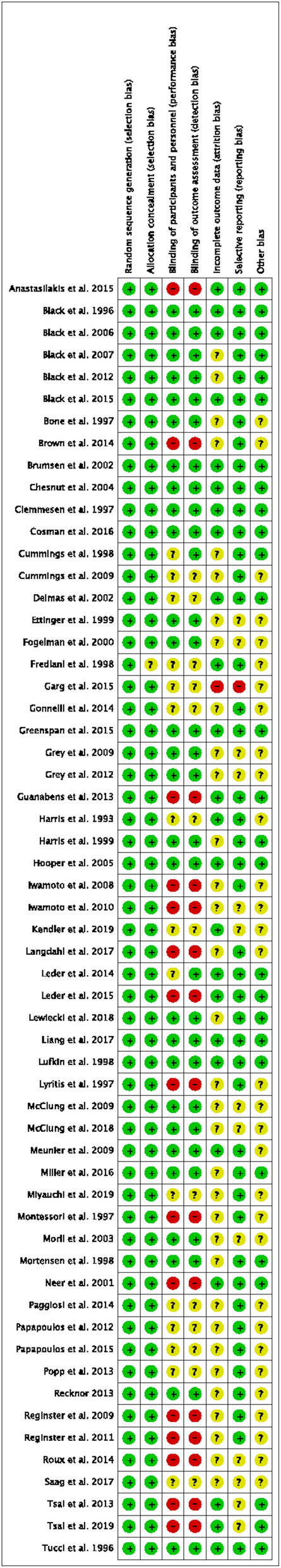
Methodological quality assessment

The risk of bias summary evidenced some point of strength of the present study. First, the randomised design of all the included studies leads to low risk of selection bias. Moreover, most studies performed assessors, patients and personnel blinding, thus leading to a low risk of performance and detection bias. The risk of attrition and reporting bias were both low. The risk to incur in unknown/other bias was low to moderate. Concluding, the risk of bias was low, attesting to the methodological assessment of the present study is a very good quality. The score of each risk of bias item for each included study is shown in Fig. 2.

### Patient demographics

Data from 82,732 patients were retrieved. The mean follow-up was 29.7 ± 19.6 months. The mean age of the patients was 67.3 ± 6.1 years. The mean BMI was 25.0 ±1.7 kg/m^2^. The mean BMD at baseline of the spine was 0.83 ± 0.11, of the hip was 0.74 ± 0.07 and of the femoral neck was 0.63 ± 0.07 g/cm^2^. The ANOVA test found baseline comparability (*P* > 0.1) with regards to age, BMI and BMD. Studies’ generalities and patients’ demographics are shown in Table 1.

**Table 1:** Generalities and patients baseline of the included studies

Author, year	Journal	Follow-up (***months***)	Calcium daily supplement (***mg***)	Vit D daily supplement (***UI***)	Treatment	Administration	Samples (***n***)	Mean age	Mean BMI (***kg/m***^***2***^)	BMD Spine (***g/cm***^***2***^)	BMD Hip (***g/cm***^***2***^)	BMD Femur neck (***g/cm***^***2***^***)***
Anastasilakis et al. 2015 [29]	*Osteoporos Int*	12	1000	800	Denosumab	IM	32	63	28.80	0.97		
					Zoledronate	IV	26	63	28.70	0.94		
					Placebo	IV	241	57	23.73	0.92	0.65	0.63
Black et al. 2007 [30]	*New England J Med*	36	1000-1500	400-1200	Zoledronate	IV	3875	73	25.10	0.79	0.65	0.53
					Placebo	IV	3861	73	25.40	0.79	0.65	0.53
Body et al. 2002 [31]	*J Clin Endocrinol Metab*	14	1000	400-1200	Alendronate	OS	73	65	24.40	0.80		
					Teriparatide	SC	73	66	23.90	0.80		
Black et al. 1996 [32]	*The Lancet*	36	652		Alendronate	OS	1022	71	25.50	0.79		0.57
			619		Placebo	OS	1005	71	25.60	0.79		0.56
Black et al. 2006 [33]	*JAMA*	60	655		Alendronate	OS	329	73	25.70	0.90	0.73	0.62
			667		Alendronate	OS	333	73	25.90	0.89	0.73	0.61
			635		Placebo	OS	437	74	25.80	0.90	0.72	0.61
Black et al. 2012 [34]	*J Bone Min Res*	36	1000-1500	400-1200	Zoledronate	IV	616	76	25.30	0.81	0.69	0.56
					Placebo	IV	617	76	25.60	0.82	0.69	0.57
Black et al. 2015 [35]	*J Bone Min Res*	36	1000-1500	400-1200	Zoledronate	IV	95	78	24.60		0.69	0.58
					Placebo	IV	95	78	25.00		0.71	0.58
Bone et al. 1997 [36]	*J Clin Endocrinol Metab*	24	813		Alendronate	OS	86	71				
			880		Alendronate	OS	89	70				
			831		Alendronate	OS	93	71				
			900		Placebo	OS	91	71				
Brown et al. 2014 [37]	*Osteoporos Int*	12			Denosumab	SC	852	68				
					Ibandronate	OS	851	67				
					Risedronate	OS						
Brumsen et al. 2002 [38]	*J Bone Min Res*	60	500	400	Pamidronate	OS	26	66		0.76		0.64
					Placebo	OS	27	64		0.74		0.64
Chesnut et al. 2004 [39]	*J Bone Min Res*	36	500	400	Ibandronate	OS	977	69	26.20			
					Ibandronate	OS	977	69	26.20			
					Placebo	OS	975	69	26.20			
Clemmesen et al. 1997 [40]	*Osteoporos Int*	36	1000		Risedronate	OS	44	67	25.50	0.80		0.61
					Risedronate/placebo	OS	44	68	24.40	0.79		0.61
					Placebo	OS	44	70	25.10	0.75		0.61
Cosman et al. 2016 [10]	*New England J Med*	12	500-1000	600-800	Romosozumab	SC	3589	71				
					Placebo	SC	3591	71				
		24	500-1000	600-800	Denosumab	SC	3589	71				
					Denosumab	SC	3591	71				
Cummings et al. 1998 [41]	*JAMA*	48	634		Alendronate	OS	2214	68	24.90	0.84		0.59
			638		Placebo	OS	2218	68	25.00	0.84		0.59
Cummings et al. 2009 [42]	*New England J Med*	36	1000	400-800	Denosumab	SC	3902	72	26.00			
					Placebo	SC	3906	72	26.00			
Delmas et al. 2002 [43]	*J Clin Endocrinol Metab*	48	500	400-600	Raloxifene	OS	2259	66	25.30	0.82		0.62
					Raloxifene	OS	2277	66	25.20	0.81		0.62
					Placebo	OS	2292	67	25.30	0.81		0.62
Ettinger et al. 1999 [7]	*JAMA*	36	500	400-600	Raloxifene	OS	2259	67				
					Raloxifene	OS	2277					
					Placebo	OS	2292					
Fogelman et al. 2000 [44]	*J Clin Endocrinol Metab*	24	1000		Risedronate	OS	184	65	24.80	0.73		0.63
					Risedronate	OS	177	65	24.80	0.75		0.64
					Placebo	OS	180	64	25.50	0.74		0.64
Frediani et al. 1998 [45]	*Clin Drug Invest*	24			Alendronate	OS	30	63	20.90	0.81		
					Calcitriol	OS	30	63	21.80	0.81		
					Alendronate/calcitriol	OS	30	63	21.00	0.80		
					Calcium	OS	30	63	21.20	0.80		
Garg et al. 2015 [46]	*J South Asian Feder Menopause* *Soc*	12			Zoledronate	IV	50					
					Teriparatide	SC	50					
Gonnelli et al. 2014 [47]	*Bone*	12	841	400	Zoledronate	IV	30	66	26.10	0.82	0.79	
			870		Ibandronate	IV	30	67	25.70	0.82	0.79	
Greenspan et al. 2015 [48]	*JAMA*	24	807	163	Zoledronate	IV	89	85	28.20	0.93	0.68	0.61
			763	168	Placebo	IV	92	86	26.90	0.97	0.70	0.62
Grey et al. 2009 [49]	*J Clin Endocrinol Metab*	24	935		Zoledronate	IV	25	62		1.06	0.85	
			916		Placebo	IV	25	65		1.03	0.86	
Grey et al. 2012 [50]	*J Clin Endocrinol Metab*	12	960		Zoledronate	IV	43	64		1.01	0.85	
			880		Zoledronate	IV	43	66		1.03	0.84	
			850		Zoledronate	IV	43	66		1.05	0.84	
			950		Placebo	IV	43	65		1.03	0.87	
Guanabens et al. 2013 [51]	*Hepatology*	24	1000		Ibandronate	OS	14	65	26.60	0.90	0.84	0.79
					Alendronate	OS	19	63	26.60	0.88	0.81	0.77
Harris et al. 1993 [52]	*Am J Med*	48	500		Phosphate-etidronate	OS	63			0.89		0.67
					Placebo-etidronate	OS	65			0.87		0.69
					Phosphate-placebo	OS	62			0.87		0.67
					Placebo	OS	63			0.86		0.68
Harris et al. 1999 [53]	*JAMA*	36	1000	500	Risedronate	OS	817	69	26.60	0.84		0.60
					Risedronate	OS	821	69	26.60	0.83		0.59
					Placebo	OS	820	68	26.50	0.83		0.60
Hooper et al. 2005 [54]	*Climacteric*	24			Risedronate	1OS	128	53		1.08		
					Risedronate	OS	129	53		1.08		
					Placebo	OD	126	53		1.08		
Iwamoto et al. 2008 [55]	*Yonsei Med J*	12	800		Alendronate	OS	61	70	21.90	0.62		
					Raloxifene	OS	61	69	21.70	0.65		
Kendler et al. 2019 [56]	*Osteoporosis Int*	12	> 1000	> 800	Romosozumab	SC	16	69				
					Romosozumab	SC	19	68				
					Romosozumab	SC	14					
					Romosozumab	SC	12					
Langdahl et al. 2017 [57]	*The Lancet*	12	500-1000	600-800	Romosozumab	SC	198	72				
					Teriparatide	SC	200	71				
Leder et al. 2015 [58]	*The Lancet*	48			Teriparatide-denosumab	SC	27	66	25.50	0.82		0.64
					Denosumab-teriparatide	SC	27	65	23.80	0.86		0.64
					Combined-denosumab	SC	23	65	25.90	0.85		0.64
Leder et al. 2014 [59]	*J Clin Endocrinol Metab*	24			Teriparatide	SC	31	66	25.50	0.82		0.64
					Denosumab	SC	33	66	24.10	0.87		0.64
					Combined	SC	30	66	25.40	0.86		0.64
Lewiecki et al. 2018 [60]	*J Clin Endocrinol Metab*	12			Denosumab	SC	3003	71	24.70			
					Denosumab	SC	3042	71	24.70			
Liang et al. 2017 [61]	*Orthop Surg*	24			Zoledronate	IV	155	57	21.80	0.63	0.75	
					Placebo	IV	95	57	21.60	0.63	0.75	
					Placebo	OS	355	64	24.10			
Lufkin et al. 1998 [62]	*J Bone Min Res*	12			Raloxifene	OS	48	67	24.80	0.75	0.64	
					Raloxifene	OS	47	67	26.20	0.81	0.69	
			750	400	Calcium/vit D	OS	48	68	25.30	0.77	0.67	
Lyritis et al. 1997 [63]	*Clin Rheumatol*	48	500		Etidronate	OS	39	72	27.60	0.57		0.42
					Calcium/vit D	OS	35	72	26.80	0.57		0.43
McClung et al. 2014 [64]	*New England J Med*	12	1000	800	Romosozumab	SC	44	67				
					Romosozumab	SC	46	67				
					Romosozumab	SC	49	67				
					Romosozumab	SC	52	67				
					Romosozumab	SC	53	67				
					Alendronate	OS	47	67				
					Teriparatide	SC	46	67				
					Placebo	SC	47	67				
McClung et al. 2009 [65]	*ObstetGynecol*	24	500-1200	400-800	Zoledronate	IV	181	60	26.50	0.86		0.69
					Zoledronate-placebo	IV	154	60	27.30	0.86		0.69
					Placebo	IV	188	61	27.20	0.86		0.69
McClung et al. 2018 [66]	*J Bone Min Res*	12	1000	800	Denosumab	SC	127	67				
					Placebo	SC	131	67				
Meunier et al. 2004 [67]	*New England J Med*	36	1000	400-800	Strontium ranelate	OS	719	69	26.20	0.73	0.69	0.59
					Placebo	OS	723	69	26.20	0.72	0.68	0.59
Meunier et al. 2009 [68]	*Osteoporos Int*	12	1000	400-800	Strontium ranelate	OS	221	72		0.85		0.66
					Strontium ranelate	OS	434	72		0.72		0.58
					Placebo	OS	225	72		0.86		0.64
Miller et al. 2016 [69]	*J Clin Endocrinol Metab*	12	1000	800	Denosumab	SC	321	69	24.30			
					Zoledronate	IV	322	70	24.30			
Miyauchi et al. 2019 [68]	*Arch Osteoporos*	36	500-1000	600-800	Denosumab	SC	247	71	21.10			
					Denosumab	SC	245	70	21.40			
Montessori et al. 1997 [70]	*Osteoporos Int*	36			Etidronate	OS	40	62		0.68	0.67	0.60
					Calcium	OS	40	63		0.67	0.69	0.61
Morii et al. 2003 [71]	*Osteoporos Int*	13			Raloxifene	OS	90	65	21.50	0.66		
					Raloxifene	OS	93	65	21.90	0.67		
					Placebo	OS	97	64	22.00	0.64		
Mortensen et al. 1998 [72]	*J Clin Endocrinol Metab*	36	937		Risedronate	OS	37	52		0.93		0.74
			1057		Risedronate	OS	38	51		0.93		0.71
			936		Placebo	OS	36	51		0.96		0.74
Neer et al. 2001 [73]	*New England J Med*	24	1000	400-1200	Teriparatide	SC	444	69		0.82	0.70	0.64
					Teriparatide	SC	434	70		0.82	0.70	0.64
					Placebo	SC	448	69		0.82	0.71	0.64
Paggiosi et al. 2014 [74]	*Osteoporos Int*	24	1200	800	Alendronate	OS	57	68	25.90	0.79	0.75	0.64
					Ibandronate	OS	58	67	26.40	0.80	0.78	0.64
					Risedronate	OS	57	67	26.80	0.81	0.80	0.67
					Control		226	38	25.10	1.,07	0.97	0.86
Papapoulos et al. 2012 [75]	*J Bone Min Res*	24			Denosumab	SC	2343	75				
					Denosumab	SC	2207	75				
Papapoulos et al. 2015 [76]	*Osteoporos Int*	60	> 1000	> 400	Denosumab	SC	2343	79				
					Denosumab	SC	2207	79				
Popp e t al. 2013 [77]	*Maturitas*	36	1000-1500	400-1200	Zoledronate	IV	55	77	24.60	0.77	0.67	0.56
					Placebo	IV	55	77	24.40	0.77	0.67	0.55
Recknor et al. 2013 [26]	*ObstetGynecol*	12	500	800	Denosumab	SC	417	67	25.50			
					Ibandronate	OS	416	66	25.10			
Reginster et al. 2009 [78]	*Osteoporos Int*	36	500-1000	400-800	Strontium ranelate	OS	879	79	25.90	0.93	0.73	0.61
					Control	OS	892	74	25.90	0.77	0.67	0.57
Reginster et al. 2011 [79]	*Osteoporos Int*	60	500-1000	400-800	Strontium ranelate	OS	233	77	25.80	0.76	0.69	0.58
					Placebo	OS	458	76	25.20			
Roux et al. 2014 [80]	*Bone*	12	≥ 1000	≥ 800	Denosumab	SC	435	68				
					Risedronate	OS	435	68				
Saag et al. 2017 [11]	*New England J Med*	24			Alendronate	OS	2047	74	25.40			
					Romosozumab-alendronate	SC-OS	2046	74	25.50			
Tsai et al. 2013 [81]	*The Lancet*	12			Teriparatide	SC	31	66	25.50	0.82	0.76	0.64
					Denosumab	SC	33	66	24.10	0.87	0.77	0.64
					Teriparatide/denosumab	SC	30	66	25.40	0.86	0.76	0.64
Tsai et al. 2019 [82]	*The Lancet*	15			Teriparatide-denosumab	SC	35	66	23.00	0.83	0.74	0.65
					Teriparatide-denosumab	SC	34	67	22.80	0.79	0.74	0.62
Tucci et al. 1996 [83]	*Am J Med*	36	500		Alendronate	OS	98	67	23.90			
					Alendronate	OS	94	64	23.30			
					Alendronate	OS	94	64	23.70			
					Placebo	OS	192	64	23.80			
Jiang et al. 2003 [84]	*J Bone Min Res*	19	1000	400-1200	Teriparatide	SC	18	68		0.77		0.61
					Teriparatide	SC	14	68		0.84		0.62
					Placebo	SC	19	68		0.86		0.65

### Outcomes of interest

**Fig. 3:**
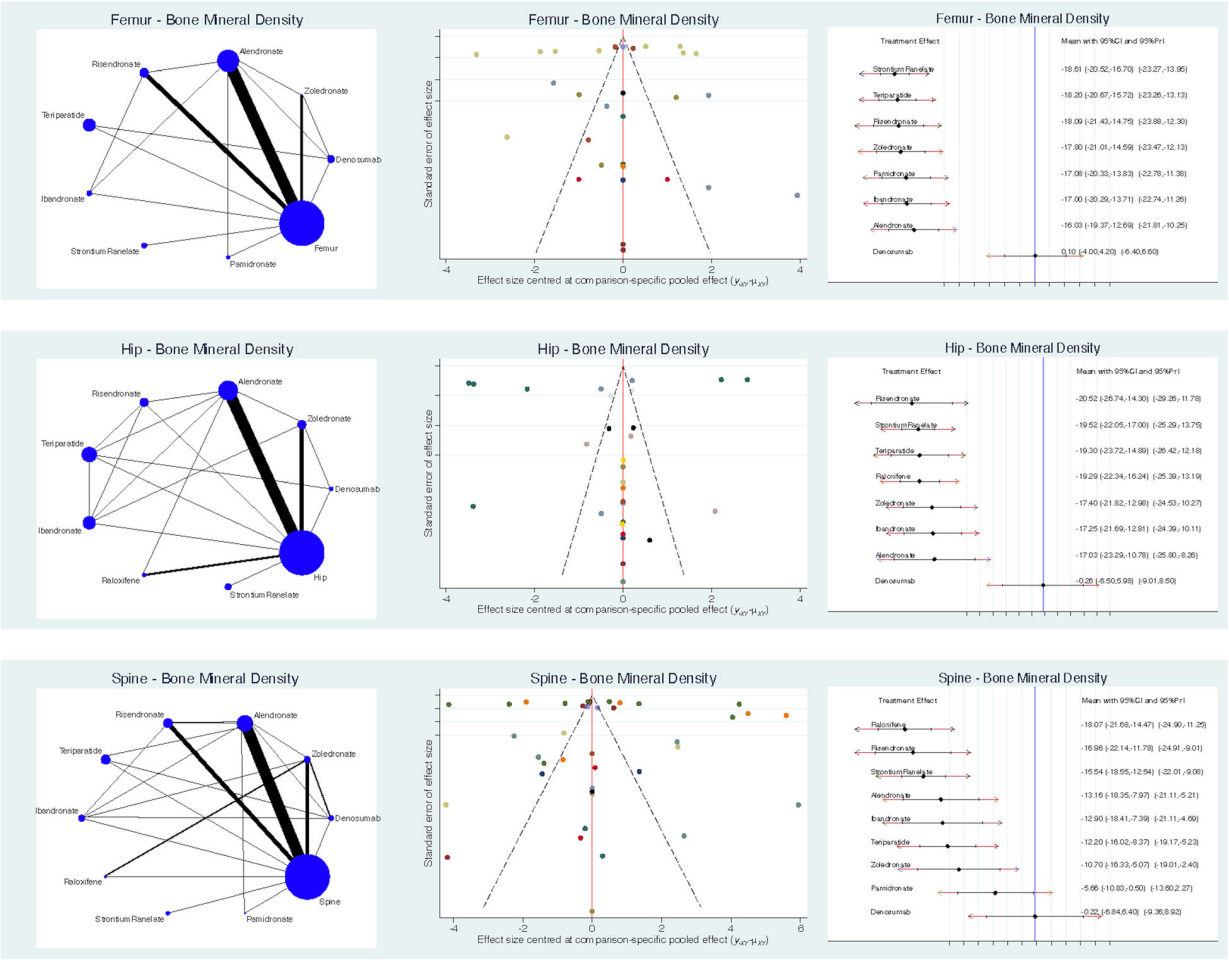
Edge, funnel and interval plots of the comparisons

Denosumab resulted in a higher spine BMD (SMD −0.22; SE 3.38; 95% CI −6.84 to 6.40), followed by pamidronate (SMD −5.66; SE 2.64; 95% CI −10.83 to −0.50) and zoledronate (SMD −10.70; SE 2.87; 95% CI −16.33 to −5.07). Denosumab resulted in a higher hip BMD (SMD −0.26; SE 3.18; 95% CI −6.50 to 5.98), followed by alendronate (SMD −17.03; SE 3.19; 95% CI −23.29 to −10.78) and ibandronate (SMD −17.25; SE 2.26; 95% CI −21.69 to −12.81). Denosumab resulted in a higher femur BMD (SMD 0.10; SE 2.09; 95% CI −4.00 to 4.20), followed by alendronate (SMD −16.03; SE 1.70; 95% CI −19.37 to −12.69) and ibandronate (SMD −17.00; SE 1.68; 95% CI −20.29 to −13.71). The equation for global linearity found no statistically significant inconsistency (*P* > 0.05) in all comparisons. Edge, funnel and interval plots of these comparisons are shown in Fig. 3.

## Discussion

Over the last decades, effective pharmaceutical treatments have been developed for the management of osteoporosis. However, most studies have not included multiple active comparators because of cost constraints, ethical problems and government regulations. This network meta-analysis is the first to include 64 RCTs with a total of 82,732 patients, including only studies with levels of evidence 1 and 2. This study compared and evaluated the influence of currently available pharmacological treatments for osteoporosis with one another in terms of BMD. The present investigation shows that denosumab was associated with the highest BMD of all evaluated osteoporosis drugs in selected women with postmenopausal osteoporosis.

Meta-analyses are considered valuable tools to analyse different studies. However, they only allow a pair-wise assessment of treatments. In contrast, network meta-analyses allow to blend together information over a network of comparisons to compare the relative effects of different treatments used for the same condition. Network meta-analysis provides vital clinical information by ranking the relative efficacy of all interventions, even those which have not been compared with one another directly.

Most previous network meta-analyses have investigated the effects of osteoporosis treatments on fracture risk, which is in contrast to our analysis which instead focused on the influence of drugs on BMD. A recent network meta-analysis of 22 RCTs studied the relative efficacy of 10 osteoporosis drugs in postmenopausal women at high risk of fragility fractures [14]. Abaloparatide had the highest probability of preventing vertebral, non-vertebral, and wrist fractures compared to placebo and all other treatment options. This was also confirmed by another network meta-analysis of 67,524 patients: both abaloparatide and teriparatide significantly reduced the fracture risk compared to placebo and other osteoporosis medications [15]. In addition, a further network meta-analysis confirmed that teriparatide seemed to be most effective in preventing new non-vertebral fractures in patients with osteoporosis [16]. A systematic review and network meta-analysis of RCTs evidenced that non-bisphosphonate interventions (including denosumab, raloxifene, teriparatide, romosozunab) are clinically effective in reducing vertebral fractures compared to placebo, and that they are beneficial for change in femoral neck BMD [17]. Romosozunab, followed by alendronate, resulted in the greatest effect on femoral BMD.

Previous studies suggest that anabolic osteoporosis treatments, such as abaloparatide and teriparatide, exert the highest influence on reducing the overall fracture risk. The present study shows that denosumab has the greatest effect on BMD, independent of the fracture risk. Denosumab demonstrates a high affinity and specificity to the RANKL, and therefore prevents it from binding to the RANKL receptors on osteoclasts and their precursors, with a direct effect on the activity and life span of existing osteoblasts [18]. Denosumab increases BMD by inhibiting bone resorption and remodelling [19]. The FREEDOM trial confirmed that denosumab, administrated every 6 months, significantly reduces the hip fracture risk by 40%, the non-vertebral fracture risk by 20% and the vertebral fracture risk by 68% [20].

The extension of the FREEDOM study showed that treatment with denosumab up to 10 years results in a cumulative gain in BMD of 21.7% at the lumbar spine, and 9.2% at the total hip, compared to baseline [21]. Denosumab resulted in lower rates of new vertebral and non-vertebral fractures throughout the study period [21]. Denosumab is administrated subcutaneously every 6 months, and therefore it is likely that the adherence to the medication is better compared to BP. This was confirmed by Kendler et al., who showed greater satisfaction when patients transitioned to denosumab as compared to a monthly oral BP [22]. Palacios et al. also confirmed a higher adherence of patients to denosumab compared to BP, and that most patients do prefer denosumab over BP for the treatment of osteoporosis [23]. The advantages of denosumab over BP seem the more favourable side-effect profile (low rates of infections and malignancies), and, as shown in the present study, the more pronounced beneficial effects on BMD. This was also confirmed previously, with denosumab more effective than ibandronate and alendronate [24–28].

Limitations of this network meta-analysis include the focus on the effects of osteoporosis treatments on spinal and hip BMD without an assessment of fracture risk reduction, adverse events or costs. The investigation of adverse effects seems to be particularly important, since adverse effects can affect adherence to treatment. Also, we only included studies which evaluated the effects of anti-osteoporosis medications for postmenopausal osteoporosis, but not for age-related, senile, or secondary osteoporosis. Further studies are necessary to examine these aspects. The minimum follow-up for a study to be included in the present network meta-analysis was 1 year. However, osteoporosis requires long-term treatment to produce clinically relevant benefits. This is especially important when certain medications, such as denosumab, have to be discontinued, and thereby lead to a potential increase in fracture risk. Another potential limitation is related to the limited variety of drugs included for analysis. Given the lack of studies in the literature, some commonly used medications, such as abaloparatide and romosozumab, were not included in the analyses. In light of these limitations, data from the present Bayesian network meta-analysis must be interpreted with caution.

Strengths of our study are the comprehensive literature search of multiple databases in multiple languages, which led to the inclusion of 64 evidence levels I and II RCTs with a total of 82,732 interventions. We also performed a rigorous review process, which was performed by two independent reviewers. Finally, we summarised and analysed the latest evidence of anti-osteoporosis medications on BMD in postmenopausal women from RCTs with the highest levels of evidence, which to our knowledge has not been performed before.

## Conclusion

The present network meta-analysis shows that denosumab followed by pamidronate and zoledronate is associated with higher spine BMD in selected women with postmenopausal osteoporosis. Denosumab followed by alendronate and ibandronate had the highest influence on hip and femoral BMD. Future studies should evaluate the effects of anti-osteoporosis drugs on the overall fracture risk and on other types of osteoporosis.

## Data Availability

This study does not contain any third material.

## Abbreviations

BMD: Bone mineral density
RANK-ligand: Receptor activator of nuclear factor-kappa B ligand
SERM: Selective oestrogen receptor modulators
BP: Bisphosphonates
PTHR1: Teriparatide
RCTs: Randomised controlled trials
SMD: Standardised mean difference
ANOVA: Analysis of variance
STD: Standardised mean difference
SE: Standard error
CI: Confidence interval
PrI: Percentile interval
BMI: Body mass index
